# Plant-Bacteria Association and Symbiosis: Are There Common Genomic Traits in *Alphaproteobacteria*?

**DOI:** 10.3390/genes2041017

**Published:** 2011-11-29

**Authors:** Francesco Pini, Marco Galardini, Marco Bazzicalupo, Alessio Mengoni

**Affiliations:** Department of Evolutionary Biology, University of Florence, via Romana 17, 50125 Firenze, Italy; E-Mails: francesco.pini@unifi.it (F.P.); marco.galardini@unifi.it (M.G.); marco.bazzicalupo@unifi.it (M.B.)

**Keywords:** bacterial genomes, plant, symbiosis

## Abstract

*Alphaproteobacteria* show a great versatility in adapting to a broad range of environments and lifestyles, with the association between bacteria and plants as one of the most intriguing, spanning from relatively unspecific nonsymbiotic association (as rhizospheric or endophytic strains) to the highly species-specific interaction of rhizobia. To shed some light on possible common genetic features in such a heterogeneous set of plant associations, the genomes of 92 *Alphaproteobacteria* strains were analyzed with a fuzzy orthologs-species detection approach. This showed that the different habitats and lifestyles of plant-associated bacteria (soil, plant colonizers, symbiont) are partially reflected by the trend to have larger genomes with respect to nonplant-associated species. A relatively large set of genes specific to symbiotic bacteria (73 orthologous groups) was found, with a remarkable presence of regulators, sugar transporters, metabolic enzymes, nodulation genes and several genes with unknown function that could be good candidates for further characterization. Interestingly, 15 orthologous groupspresent in all plant-associated bacteria (symbiotic and nonsymbiotic), but absent in nonplant-associated bacteria, were also found, whose functions were mainly related to regulation of gene expression and electron transport. Two of these orthologous groups were also detected in fully sequenced plant-associated *Betaproteobacteria* and *Gammaproteobacteria.* Overall these results lead us to hypothesize that plant-bacteria associations, though quite variable, are partially supported by a conserved set of unsuspected gene functions.

## 1. Introduction

The phylum *Proteobacteria* is the most numerous group currently recognized in the domain Bacteria [1]. Within this group, the class of *Alphaproteobacteria* harbors a miscellaneous set of metabolisms, cellular phenotypes and a wide range of habitats, including phototrophic genera (*Rhodobacter*), symbionts of plants (*Rhizobium*, *Sinorhizobium*, *Mesorhizobium* and *Azorhizobium* [2]), animal and plant pathogens (*Rickettsia*, *Brucella*, *Agrobacterium*) and also genera able to metabolize C1 compounds (*Methylobacterium*). In addition, mitochondria have a common origin with SAR11 clade, as a sister group of the order *Rickettsiales* [3]. Habitats that are colonized by *Alphaproteobacteria*, range from the ocean floor volcanic environments, to soil, in which they may interact with plant roots, to surface waters of oceans [1].

*Alphaproteobacteria*, with nearly 600 completely sequenced genomes, is one of the most studied bacterial classes [1], showing a large heterogeneity in genome size, from 1.1 to 9.2 Mbp [4] and genome architecture, with the presence of additional replicons, such as chromids [5], and plasmids [6]. Because of these genomic traits, and also thanks to their versatility in adapting to different habitats, *Alphaproteobacteria* constitute an excellent model system to study how bacterial genomes evolve and how genomic features are related to environmental adaptation [1,4].

Particularly intriguing is the alphaproteobacterial ability to interact with plants, as pathogens and as nonpathogenic mutualist/commensals (symbionts/nonsymbionts) (e.g., *Rhizobium*, *Azospirillum*). Plant-associated bacteria *sensu lato* can be found in, and around roots, in the vasculature, and on aerial tissues or in specifically developed organs (e.g., root nodules) [7], allowing to categorize strains as phyllospheric, rhizospheric and endophytic.

Phyllospheric bacteria inhabits the aerial parts of the plant (leaves, stems, buds, flowers and fruits), possibly affecting plant fitness and productivity of agricultural crops [8]. The rhizosphere is the part of soil around plant roots populated by microbes (bacteria and fungi); microorganisms from the rhizosphere interact with roots in several process such as the decomposition of organic matter, the maintenance of soil structure and water relationships, as a consequence rhizosphere is a fundamental niche of the soil ecosystem [9]. Endophytic bacteria can be defined as those bacteria that colonize the internal tissue of the plants (endosphere) with no external sign of infection or negative effect on their host [10]; they can be classified as ‘obligate’ or ‘facultative’ endophytes in accordance with their life strategies. Obligate endophytes are strictly dependent on the host plant for their growth and survival and transmission to other plants could occur only by seeds or via vectors, while facultative endophytes could grow outside host plants [11]. Finally, a noteworthy endophytic example within *Alphaproteobacteria*, is the nitrogen-fixing symbiosis established with leguminous plants by rhizobia, which is coupled with the development of a new plant structure, the nodule, in the root or in the stem of the plant [12]. All these heterogenous phenotypes suggest that it could be difficult to find common genetic traits for Plant-associated bacteria.

An additional degree of complexity is given by the fact that single species or even single strains inhabit both soil and plant tissues and can show multiple types of plant association. For example, *Azospirillum* strains are known as model plant-growth promoting rhizosphere (PGPR) bacteria, but they have also been shown within plant tissue, as endophytes of cereals [13]; on the other hand, the specific alfalfa symbiont *Sinorhizobium meliloti* is also able to grow as rhizospheric of nontarget host plants and it behaves as endophytes with cereals like rice [14], besides free-living in bulk soil. Such observations led to doubt whether a genetic common background is present within all plant-associated *Alphaproteobacteria*. In fact, concerning symbiotic species, it is fairly accepted that the symbiotic lifestyle needs some specific genetic functions (e.g., *nod* genes), which are not present in nonsymbiotic nitrogen fixers [15]. However, stem-nodulating bradyrhizobia have shown that a *nod*-independent symbiosis can be established [15,16]. Two questions therefore arise: (i) Is the symbiotic lifestyle in α-rhizobia characterized by the presence of a common gene set? (ii) Do all plant-associated species (both symbiotic and nonsymbiotic) share some common genes conferring the ability to associate with plants? One way to begin to answer these questions is to apply a comparative genomics approach. Previous investigations on the comparison of α- and β-rhizobia have been performed [15,17] as well as the comparison of some Plant-associated endophytes in *Gammaproteobacteria* [18], however no systematic analyses have been attempted in *Alphaproteobacteria*.

Here we report a bioinformatic analysis aimed at the scanning of all the alphaproteobacterial sequenced genomes trying to sort out the possible exclusive or distinctive genes which enable some of the *Alphaproteobacteria* to be associated with plants, evaluating if plant-bacteria association needs a specific assortment of gene functions or if, as suggested by its phenotypic heterogeneity, it is rather unrelated to the presence of a dedicated set of genes.

## 2. Results and Discussion

### 2.1. Plant-Associated Bacteria Have Larger Genomes than Nonplant-Associated

**Figure 1:**
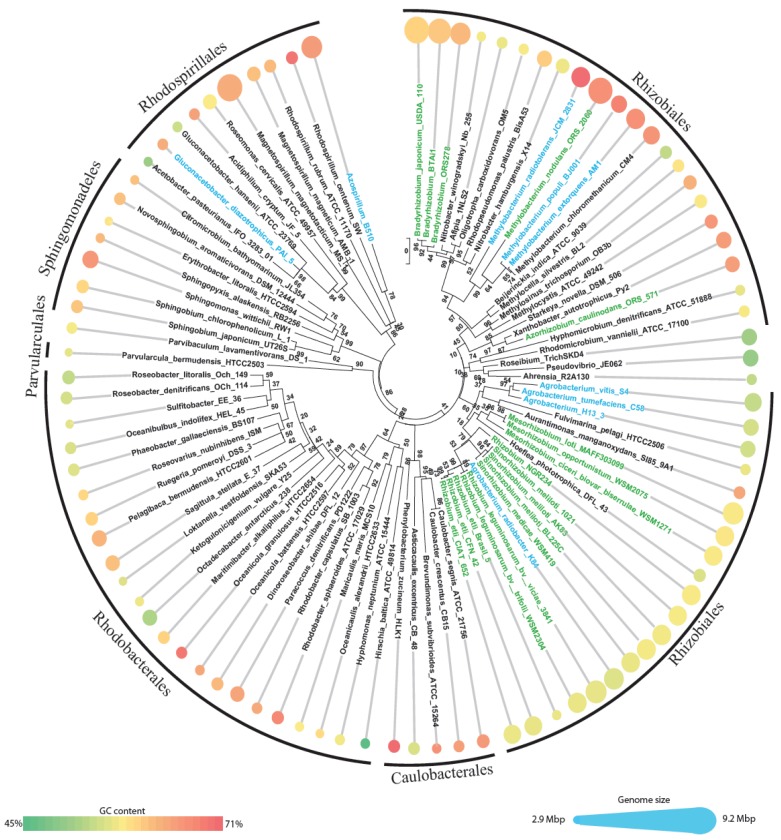
Phylogenetic tree based on 16S rRNA gene sequence for the 92 selected organisms. Names in green and cyan indicate plant-associated species (green, symbionts; cyan, nonsymbionts). The dimension of the circles is proportional to the genome size, while the color of the circles indicates the GC content.

First a dataset of the relevant *Alphaproteobacteria* (“alphas” for short) was constructed by downloading all the alphaproteobacterial genomes available in NCBI genome database. All animal obligate pathogens were excluded, since they show extensive genome reductions, linked with intracellular lifestyle [4,19], as well as the SAR11 clade due to the extensive gene loss described for this group [1]. A total of 92 genomes were then analyzed (Figure 1 and Supplementary Material S1), and divided into three groups: (i) solely free-living, (ii) plant-associated and (iii) symbiont (that is a sub-set of plant-associated) combining the information available on GOLD database [20,21], Bergey's manual of systematic bacteriology [22] and bibliographic search on Pubmed. Plant-associated bacteria include 27 genomes (2 pathogens, 7 associated and 18 symbionts), all but two (25/27) grouped within the order *Rhizobiales* (Figure 1), the only exceptions are the species *Gluconacetobacter diazotrophicus* and *Azospirillum* B510 which fall in the order *Rhodospirillales*; of course we cannot exclude that among the 65 nonplant-associated bacteria some could have also experienced the plant environment, even if those putative events have not been reported.

A quick look at these genomes shows a wide range of genome sizes, spanning from 2.9 Mbp (*Parvularcula bermudensis* HTCC2503) to 9.2 Mbp (the magnetotactic bacterium *Magnetospirillum magnetotacticum* MS1). The average genome size (±standard deviation) of the dataset is 5.04 ± 1.53 Mbp. Plant-associated bacteria have significant (*P* < 0.0001, one-way ANOVA) larger genomes (6.73 ± 1.26 Mbp) than nonplant-associated ones (4.34 ± 0.99 Mbp), as previously noticed [19]. The same trend was observed considering only the order *Rhizobiales*, which accounts for near half of the entire dataset (42 out of 92 genomes with average length of 5.83 ± 1.53 Mbp), with plant-associated *Rhizobiales* having genomes larger than those of nonplant-associated *Rhizobiales* (6.81 Mbp and 4.47 Mbp, respectively, P < 0.0001). Average GC content is 63.1 ± 4.4% and is similar for plant-associated and nonplant-associated genomes (63.03% and 62.71%, respectively, *P* < 0.8) and ranges from 45.2% (*Hirschia baltica* ATCC49814) to 71.1% (*Phenylobacterium zucineum* HLK1); within the *Rhizobiales* we observed the same trend: an average of 63.1% with 63.0% for plant-associated and 63.1% for nonplant-associated).

### 2.2. Are There Life-Style Specific Genes in Alphaproteobacteria?

**Figure 2:**
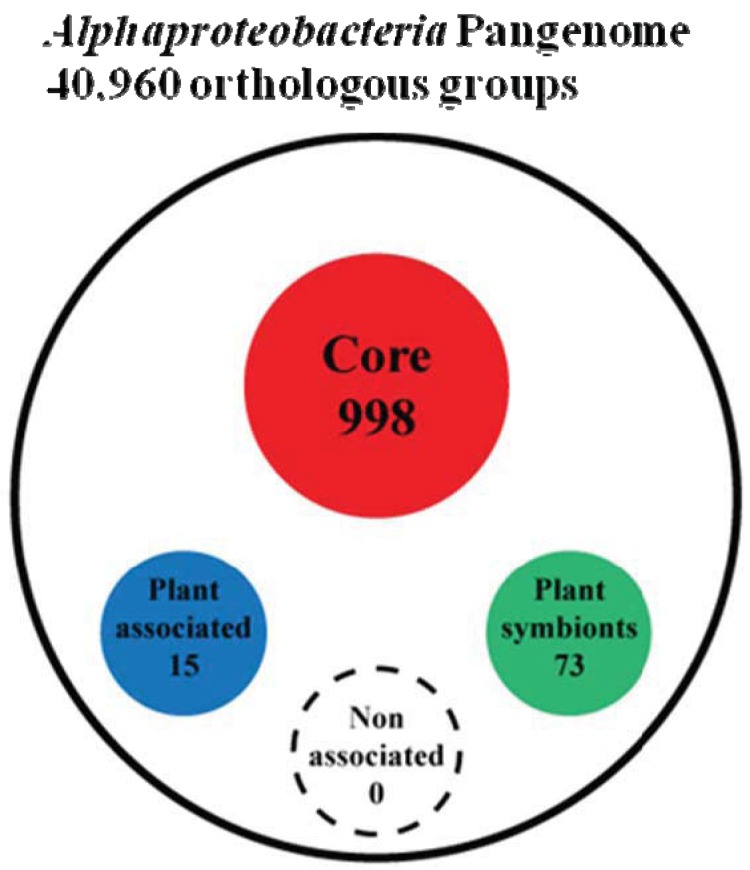
Number of orthologous groups found inside each life-style species list. Circles sizes are not in scale.

To answer this question we first peformed a genome clusterization of all protein coding genes present in the 92 genomes, obtaining 40,960 groups of orthologs (out of a total number of 434,411 proteins analyzed). Next, starting from these groups of orthologs, we proceeded trying to extract four groups named as: (i) Alpha Core (common to all the analyzed organisms), (ii) Plant-Associated (common to and exclusive of plant associated bacteria), (iii) Plant-Symbionts (common to and exclusive of plant symbionts), and (iv) NonPlant-Associated (common to and exclusive of nonplant-associated bacteria). Since genetic elements inside bacteria are prone to horizontal gene transfer and therefore genes that may be specific for a certain life-style may be found also in other bacterial species, by chance or because they might carry out a different function, we developed an orthologs-species clustering approach capable of taking into account this dynamical behavior. This “Fuzzy orthologs-species clusterization” analysis (see Materials and Methods) sorted out 998 orthologous groups for the Alpha Core subset, while life-style specific subset of NonPlant-Associated, Plant-Associated and Plant-Symbionts accounted for 88, 15 and 73 orthologous groups, respectively (Figure 2 and Supplementary Material S2). As expected, the Non-Plant subset of orthologous groups was found to be inconsistent, since a series of random subsets having the same number of species (see materials and methods) showed a similar number of orthologous groups. This finding is not surprising, since the NonPlant subset consists of species with no unique distinctive habitat, varying from soil to marine and freshwater organisms. On the contrary, for the other subsets, the number of orthologous groups generated from random species list was zero; the same result was observed when sampling only inside the order *Rhizobiales* (where most of the Plant-associated species are), retrieving 1.7 orthologous groups on average (data not shown). Notably, when we did not apply fuzziness to the orthologs-species clusterization, we did not find anyorthologous groups in any subset, but only in the Alpha Coreone. This discrepancy suggests that the plant association behavior may not be dependent on a strongly conserved defined set of genes strictly common to all the species of the subset. However, interestingly, by applying the “Fuzzy orthologs-species clusterization”, the larger Plant-Associated subset was found to contain only 15 orthologous groups, while in contrast the smaller Plant Symbionts subset contains 73 orthologous groups. This finding again confirms that the generic plant association behavior does not require a large repertoire of specific genes, while the symbiotic interaction in α-rhizobia is more dependent on the presence of specific gene traits [23].

### 2.3. Which Biological Functions Are Encoded by Life-Style Associated Orthologous Groups?

**Figure 3:**
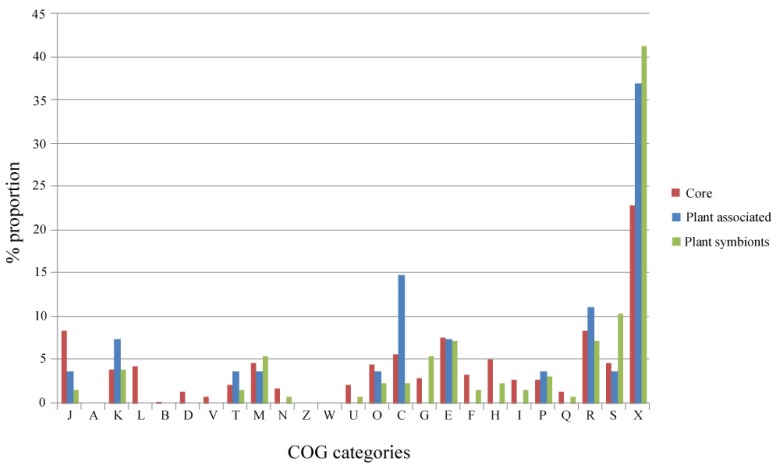
Percent distribution of orthologous groups belonging to the different subsets (Core, Plant-Associated and Plant symbionts) among Cluster of Orthologous Groups (COG) categories. Note that each orthologous group can be mapped to more than one category. The list of COG codes is reported in Supplementary Material S3.

An overview of the distribution among the COGs (Cluster of Orthologous Groups) categories of the genes present in the Alpha Core, Plant-Associated and Plant Symbionts subsets is reported in Figure 3. Regarding the Plant-Associated and the Plant Symbionts subsets, COG categories related to basic cell functions (L, Replication, recombination and repair; B, Chromatin structure and dynamics D, Cell cycle control, cell division, chromosome partitioning; V, Defense mechanisms) are not represented, since they are mostly present in the Alpha Core subset, as expected; on the other hand, COG categories poorly or not characterized (S and X), are the most represented in both plant related subsets. The categories related to regulation of gene expression (K and J) and energy production and conversion (C) show a slightly higher proportion in the Plant-Associated subset. Regarding the Plant Symbionts subset, a slightly higher over-representation of the carbohydrate transport and metabolism category (G) was observed, suggesting the key role of carbohydrate metabolism for establishing nitrogen fixing symbiosis (*i.e*., for the formation of the so-called Nod factors as well as for bacteroid trophism [23]). Indeed, some plant symbionts, as for instance *Sinorhizobium meliloti*, contain large genomic regions or replicons mainly devoted to carbohydrate transport and metabolism [24-26]. However, the percentages of COG categories represented in the different subsets are not statistically different (Spearman Rank Correlation and Chi-square test with Monte Carlo simulation, data not shown).

**Figure 4:**
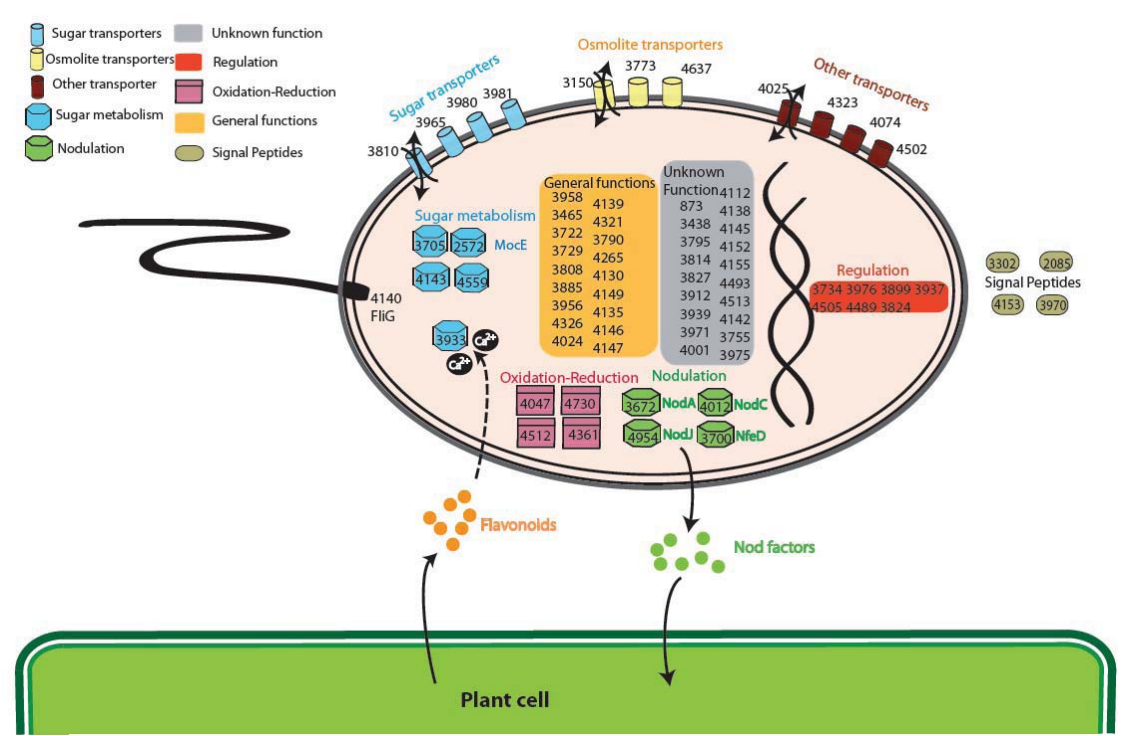
Overview of the cellular functions of the Plant Symbionts gene set. Go categories are color coded. Numbers represent orthologous groups (see Supplementary Material S2).

The analysis of the GO (Gene Ontology) categories in the Plant-Associated subset (Supplementary Material S3) shows that the most represented biological process is related to electron carrier activity (4 groups), while for the Plant Symbionts the functions encoded appear to be more heterogeneous (Figure 4). In particular, proteins involved in many process were found, ranging from symbiosis specific functions, like nodulation (4 orthologous groups: 3672, 4012 and 4954 encoding NodA, NodC, and the NodJ protein respectively, plus group 3700 also encoding NfeD another protein necessary for nodulation [27]) to trascriptional regulation and to more general biological functions (especially oxidation-reduction), with a slightly higher presence of transport-related functions (11 groups, with 3 of them probably involved in osmolarity control). As reported in Figure 4, sugar transport (4 groups out of 11 transporter) and metabolism (5 groups), are highly represented in the symbiont subset. Within this category, of particular interest is group 2572 encoding for MocE, a Rieske non-heme iron oxygenase essential in the catabolism of rhizopines (3-*O*-methyl*scyllo*-inosamine, 3-*O*-M*S*I) a nodule-specific compounds that confer an intraspecies competitive nodulation advantage to strains able to utilize them [28]. Another intriguing group is 3933 which encodes for a protein belonging to the senescence marker protein 30 (SMP-30)/gluconolaconase superfamily which contains many mammalian sequences [29]; this protein was found to accommodate multiple functions [30], among which calcium regulation (as a regucalcin) [31]; that is particularly intriguing as the involvement of Ca^2+^ in the symbiotic signaling pathway activated by flavonoids was found in *Rhizobium leguminosarum* bv. viciae [32]. Another interesting function which could be related to the plant symbiosis is cell motility, encoded by orthologous group 4140 (protein FliG). No nitrogen-fixation related proteins were found as exclusive, due to the presence in alphas of free-living nitrogen-fixing species (*Xanthobacter autotrophicus* [22]) and to the presence of the *fix* signaling module also in *Caulobacter* [33].

Interestingly, none of the functions previously putatively associated with endophytic life-style was detected, as type IV pili [34] or other metabolic or hormonal-related activities [35]. This is possibly due to the wide range of associations of our Plant-Associated subset, which includes also symbiotic and rhizospheric interaction; absence of type IV pili could be also linked to their involvement in many other processes in other species not engaged in plant interactions.

### 2.4. Taxonomic Range of the Life-Style Associated Genes Outside Alphaproteobacteria

**Figure 5:**
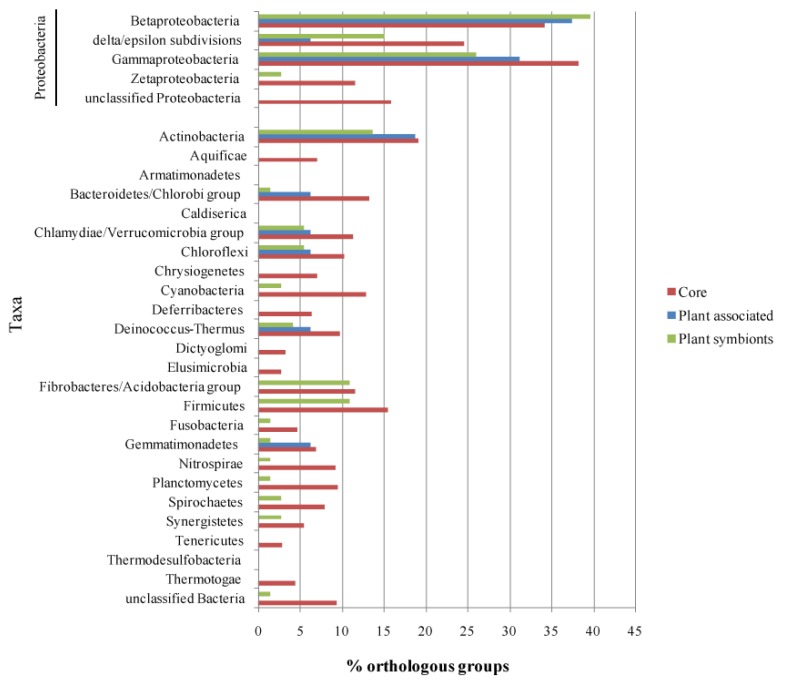
Taxonomic sharing of life-style associated genes. For each taxonomic division (according to NCBI), the proportion of the life-style related orthologous groups having at least one significant hit is shown.

Once the list of life-style related orthologous groups was defined, we looked for their presence in the other branches of the bacterial taxonomic tree, in order to understand if such functions are specific for alphas or are widespread in other taxas, thus giving an insight into the evolutionary pathways of those functions. Each life-style associated orthologous group, was then used as a query on the GenBank database to find homologous sequences in all bacterial taxa; results of the analysis are reported in Figure 5 and Supplementary Material S4. Figure 5 offers an overview of the proportion of the orthologous groups occurring in each subset which have hits in the different bacterial classes. As expected, most of the hits were scored within *Proteobacteria*, in particular in the classes *Beta* and *Gamma*, possibly reflecting both a higher phylogenetic proximity and a general bias of the database which is abundant in sequences from members of such classes (*Alphaproteobacteria* were excluded from the analysis).

A high proportion of Alpha Core genes have at least one hit in almost all the taxa probed by the analysis, with an average of 10.5% of the Alpha Core subset having an hit in the selected taxa, while on average, only 4.2% and 5.0% of the Plant Associated and Plant Symbionts orthologous groups have at least a hit in each taxa; this observation suggests that plant association genes tend to be conserved only inside *Alphaproteobacteria* and to a lesser extent inside *Beta*- and *Gamma-Proteobacteria* (36% average), while just few genes have an homolog in phylogenetically distant species, where they might not be related to a plant association behavior.

To further elucidate this point, all the taxa found by this approach were investigated for their plant-association life-style, according to the GOLD database annotation; 33.3% of the Plant-Associated orthologous groups have at least one hit in species associated with plants, followed by the Plant Symbionts (30.1%) and the Alpha Core (24.6%). The two plant related subsets have plant association hits only inside the *Protebacteria* class (*Beta-* and *Gamma-Proteobacteria*), while the Alpha Core hits are distributed in a broader range, including *Actinobacteria*, *Cyanobacteria* and *Firmicutes* (Supplementary Material S5); the results of this analysis then imply that the plant association related genes are rather specific of the *Proteobacteria* class, while the housekeeping genes exhibit an higher degree of sequence conservation across all the bacterial phylogenetic tree.

To shed some light on the possibility that Plant-associated specific genes are involved in plant association at a broader taxonomic level, the protein coding genes found as exclusively present in Plant-Associated alphas were checked for their presence in the genomes of four known and fully-sequenced plant-associated *Proteobacteria*, in particular in the class of *Beta-Proteobacteria* the strains *Cuprividus taiwanensis* [17] and *Azoarcus* sp. BH72 [34] and in the class of *Gamma-Proteobacteria* the species *Enterobacter* sp. 638 [35] and *Klebsiella pneumoniae* 342 [36]. Results of the comparison are shown in Table 1. Interestingly, two out of 15 orthologous groups are present in all the four strains selected, namely orthologous group 2149 (Transcriptional regulator) and 2774 (endoribonuclease l-psp), suggesting that regulation (either by transcriptional regulation and RNA stability) may play pivotal roles in establishing the association with plant. Moreover 5 other genes were found to be present in at least 2 of the 4 species investigated. A previous work dealing with the description of the genome sequence of the β-rhizobium *Cuprividus taiwanensis* [17], found no gene both common and specific to all rhizobia, suggesting that symbiotic association with plants evolved with multiple strategies, even though genes preferentially associated (on a statistical basis) with plant symbiosis were detected. However, our findings suggest that these two genes, putatively needed in *Alphaproteobacteria* to establish a successful interaction with plants, are also present in the *Beta* and *Gamma-Proteobacteria* model organisms for plant association. Endoribonuclease L-PSP is involved in single-stranded mRNA cleavage in *Leishmania infantum*, a parasite that in its life cycle alternates two stages, and is hypothesized to be involved in specific post-transcriptional regulation of gene expression [37]; characterization of mutants for those orthologs is however necessary to fully elucidate their role in plant association in such a broad taxonomic background.

**Table 1:** Phylogenetic conservation of plant-associated orthologous groups in other plant-associated bacteria. The hit for each genome is indicated as GenBank accession number of the corresponding protein.

**Orthologous group**	**Function**	***Azoarcus* BH72**	***Cupriavidus taiwanensis***	***Enterobacter* 638**	***Klebsiella pneumoniae***
**2149**	Transcriptional regulator	YP_932298	YP_002005188 YP_002007781	YP_001177142	YP_002237096 YP_002237759
				YP_001177763	
				YP_001177947	
**2248**	Transcriptional regulator		YP_001795747	YP_001177733	YP_002239091 YP_002240771
				YP_001177763	
				YP_001177947	
**2654**	Adenylate cyclase	YP_932132	YP_002008552		
**2734**	ABC transporter			YP_001175837	YP_002237478
				YP_001177423	YP_002239806
**2737**	Unknown		YP_002005759		
**2774**	Endoribonuclease l-psp	YP_931980	YP_002008711	YP_001178228	YP_002237662
			YP_002008874		YP_002238064
**2791**	Phosphoesterase				
**2853**	Electron transfer flavoprotein, beta subunit		YP_001796225		
**2898**	Unknown				YP_002236173
**2908**	Electron transfer flavoprotein, alpha subunit		YP_001796224		
**2912**	Methyltransferase	YP_935409		YP_001176000	YP_002237443
				YP_001177854	YP_002239590
**2927**	Aminoacid aldolase or racemase		YP_002007445		
**2981**	Mg^2+^ and Co^2+^ transporters		YP_002006900	YP_001176870	YP_002238775
					YP_002238859
**3082**	Ferredoxin-like protein		YP_001796222		
**3137**	Unknown				YP_002236905

## 3. Experimental Section

### 3.1. Phylogenetic Tree

To construct our reference phylogenetic tree, all 16S rRNA gene sequences were aligned using MUSCLE [38], alignment was manually checked. The alignment was used with the software Mega 5.05 [39] to generate a phylogenetic tree. A Model test (Supplementary Material S6) was performed before running the Maximum Likelihood algorithm, with 1,000 bootstrap replicates and the Tamura-Nei model of evolution.

### 3.2. Genomes Clusterization

The 92 genomes were clustered together using the approach proposed by Kim and collaborators [40], using the PanGenomer software (available upon request); a total number of 8,464 pairwise InParanoid analyses with no thresholds were generated and the results were merged in a single file as an input for MCL [41], using an inflation factor of 5.0, a pruning threshold of 30,000 and a selection number of 5,000. To test the clusterization the presence of 5 well known orthologous genes, involved in cell cycle regulation and DNA replication (*ctrA*, *dnaA*, *rpoE*, *gyrB*, *dnaQ*), was used as a positive control (referred to as group 4, 315, 158, 524 and 26 respectively).

### 3.3. Fuzzy Orthologs-Species Clusterization

The obtained orthologous groups were mapped to the four species subsets (Figure 1) looking at the species from which each protein belonging to that group came from, using a so-called “Fuzzy” approach: an orthologous group was regarded as specific for one of the four subsets when its species list was comprised between 80% and 110% of the subset list. The biological value of each subset was tested generating 10 random organism lists with the same length of the subset and looking at how many orthologous groups were then retrieved.

### 3.4. Orthologous Groups Annotation

The orthologous groups belonging to the four subsets were annotated, using ten proteins from each group (selected randomly) to speed-up the analysis. Each protein was mapped to the COG database [42] using rpsblast 2.2.25+ and an e-value threshold of 1e-10; the domain content and the GO [43] annotation were obtained using Iprscan 4.8 [44] with the InterPro database release 33.0.

### 3.5. Taxonomic Analysis

The protein sequence similarity across the bacterial kingdom of each orthologous group was inspected using TaxonomyBlaster (available upon request); the same proteins used for the annotation were analyzed using a series of taxonomically-restricted portions of the NCBI nr database (downloaded on 1 July 2011): all the taxonomic classes (excluding the “environmental samples”) inside the *Bacteria* kingdom were iteratively used; the *Proteobacteria* class was further divided into the distinct classes, excluding the *Alphaproteobacteria* and the Proteobacterial “environmental samples”. BLAST was run using the BLOSUM45 matrix, the soft masking option, a fixed database size of 500,000,000 and the Smith-Waterman local optimal alignments option. Those hits showing an e-value below 1e-10, a query coverage above 66% and an homology index above 0.33, were retained. The obtained species were marked as Plant-Associated looking at the available information in the GOLD database [20].

## 4. Conclusions

As vector-borne intracellular *Alphaproteobacteria* have evolved towards smaller genome [4], the trend in plant-associated *Alphaproteobacteria* seems to be headed in the opposite direction towards an increase of genome size, probably due to the different habitats colonized including soil and plant tissues [19]. Here, for the first time to our best knowledge, we report an investigation of the genomic features, as different genes, which could be related, on a genomic basis, to the symbiotic and nonsymbiotic plant-association in *Alphaproteobacteria.* This analysis was carried out with a novel orthologs-species clusterization approach that was able to take into account the natural horizontal gene transfer dynamics, allowing us to also identify those genes that are (partially) shared with other species or that are not present in all the life-style related species.

Interestingly, a relatively large set of genes shared by an exclusive symbiotic alphaproteobacterial species was found, suggesting that a common genomic base is indeed present; tough multiple “recipes” for plant association are present [15]. This set includes functions previously known to be linked to the symbiotic interaction, but also others, which were previously unsuspected. In particular, genes necessary for plant-bacteria communication were retrieved as well as an enrichment in protein coding genes involved in sugar transport and metabolism. Most of these orthologs could likely be associated with metabolic exchanges and communication between plant cells and bacteroids; interestingly an ortholog of SMP30/gluconolaconase family (regucalcin) was also found suggesting a link between nodulation and calcium spiking in the rhizobial cell, in agreement with recent experimental findings[32].

Contrary to what could be expected by this highly heterogeneous phenotype, concerning all plant-associated species obtained, results showed a numerically low, but computationally consistent, set of genes which could account for their ability to associate with plants as both symbiont and nonsymbiont (*i.e*., rhizospheric, pathogen, endophyte). Interestingly, several functions were related to the regulation of gene expression, which makes sense considering the pivotal role of the perception of environmental signals for association with plants. This set of putatively plant-associated genes showed two apparently contradictory properties: a relatively high degree of conservation of these few genes inside *Proteobacteria* (when compared to the other branches of the bacterial tree) but also a certain degree of conservation across phylogenetically distant plant-associated species. This evidence could mean that even though there are no common genetic traits that distinguish this ecologically heterogeneous group of species, single genetic “pieces” may be shared, in a vast phylogenetic range, with other plant-associated species. We can then speculate that association with plants is therefore addressed using several pathways and mechanisms (which mirror the different types of association), even within a relatively narrow taxonomic range.

In conclusion, while symbiotic lifestyle needs a defined gene set, nonsymbiotic plant-bacteria association can occur through multiple strategies with functions specific for the single interaction.

## Supplementary Material

**S1.** Alphaproteobacteria dataset.
**S2.** Clusterization of orthologous groups in the four subset analyzed (Alphas, NonPlant-Associated, Plant-Associated, Symbionts).
**S3.** COG names list, Gene Ontology and COGs categories for Plant-associated and Symbionts.
**S4.** Taxonomic sharing of life-style associated genes.
**S5.** Taxonomic sharing of life-style associated genes in other plant-associated species.
**S6.** Model test.

Supplementary File 1 ZIP-Document (ZIP, 1441 KB)
